# Potato spindle tuber viroid

**DOI:** 10.18699/VJ21.030

**Published:** 2021-05

**Authors:** A.V. Kochetov, A.Y. Pronozin, N.V. Shatskaya, D.A. Afonnikov, O.S. Afanasenko

**Affiliations:** Institute of Cytology and Genetics of the Siberian Branch of the Russian Academy of Sciences, Novosibirsk, Russia Novosibirsk State University, Novosibirsk, Russia; Institute of Cytology and Genetics of the Siberian Branch of the Russian Academy of Sciences, Novosibirsk, Russia; Institute of Cytology and Genetics of the Siberian Branch of the Russian Academy of Sciences, Novosibirsk, Russia; Institute of Cytology and Genetics of the Siberian Branch of the Russian Academy of Sciences, Novosibirsk, Russia Novosibirsk State University, Novosibirsk, Russia; Institute of Cytology and Genetics of the Siberian Branch of the Russian Academy of Sciences, Novosibirsk, Russia All-Russian Institute of Plant Protection, Pushkin, St. Petersburg, Russia

**Keywords:** viroids, plants, pathogenesis, генетика вироида, патогенез растений

## Abstract

Viroids belong to a very interesting class of molecules attracting researchers in phytopathology and molecular evolution. Here we review recent literature data concerning the genetics of Potato spindle tuber viroid (PSTVd) and the mechanisms related to its pathological effect on the host plants. PSTVd can be transmitted vertically through microspores and macrospores, but not with pollen from another infected plant. The 359 nucleotidelong genomic RNA of PSTVd is highly structured and its 3D-conformation is responsible for interaction with host cellular factors to mediate replication, transport between tissues during systemic infection and the severity of pathological symptoms. RNA replication is prone to errors and infected plants contain a population of mutated forms of the PSTVd genome. Interestingly, at 7 DAI, only 25 % of the newly synthesized RNAs were identical to the master copy, but this proportion increased to up to 70 % at 14 DAI and remained the same afterwards. PSTVd infection induces the immune response in host plants. There are PSTVd strains with a severe, a moderate or a mild pathological effect. Interestingly, viroid replication itself does not necessarily induce strong morphological or physiological symptoms. In the case of PSTVd, disease symptoms may occur due to RNA-interference, which decreases the expression levels of some important cellular regulatory factors, such as, for example, potato StTCP23 from the gibberellic acid pathway with a role in tuber morphogenesis or tomato FRIGIDA-like protein 3 with an early flowering phenotype. This association between the small segments of viroid genomic RNAs complementary to the untranslated regions of cellular mRNAs and disease symptoms provides a way for new resistant cultivars to be developed by genetic editing. To conclude, viroids provide a unique model to reveal the fundamental features of living systems, which appeared early in evolution and still remain undiscovered.

## Abstract (ru)

Вироиды, небольшие кольцевые молекулы РНК, которые вызывают патогенез у растений, остаются одним из самых необычных биологических объектов, привлекающих внимание не только фитопатологов, но и специалистов в области молекулярной эволюции. В статье приведен обзор последних литературных данных о генетике вироида веретеновидности клубней картофеля (ВВКК) и генетических механизмах формирования патологических состояний у растений-хозяев. ВВКК способен передаваться вертикально (через генеративные клетки зараженного растения), но, в отличие от некоторых других вироидов, не передается через пыльцу от зараженного растения. Большой интерес у исследователей вызывает структура геномной РНК вироида размером 359 нуклеотидов: хорошо известно, что особенности 3D конформации определяют основные параметры взаимодействия с клеточными факторами на стадии репликации, транспорта между различными тканями в процессе системной инфекции, а также степень выраженности симптомов заболевания. При репликации геномной РНК вироидов часто происходят ошибки, приводящие к появлению гетерогенной популяции молекул РНК в тканях зараженного растения. Примечательно, что через 7 дней после инокуляции только 25 % молекул геномной РНК ВВКК соответствовали исходной матрице, использованной для инокуляции, однако эта доля увеличилась до 70 % через 14 дней и далее оставалась на том же уровне. По-видимому, при сохранении у мутантных вариантов геномной РНК способности к репликации вироид обладает высоким потенциалом к отбору эффективных инфекционных форм. ВВКК вызывает у пораженных растений развитие иммунного ответа, механизмы индукции которого недостаточно изучены. Известны сильно- и слабопатогенные штаммы ВВКК, вызывающие разные проявления болезни, фенотипические проявления от которых у пораженных растений в значительной мере различны. Сама по себе репликация вироида не обязательно приводит к выраженным фенотипическим проявлениям, в случае ВВКК они могут быть связаны с участками гомологии между геномной РНК и мРНК некоторых регуляторных генов, например транскрипционного фактора StTCP23 картофеля, участвующего в регуляторном контуре гиббереллиновой кислоты и в контроле морфогенеза клубня. Другой пример – индукция РНК-интерференции против мРНК гена FRIGIDA-like protein 3 у томата, что приводит к раннему цветению. В связи с этим обсуждаются потенциальные способы борьбы с вироидом, основанные на удалении из генома растений таких участков гомологии, расположенных в нетранслируемых областях мРНК и не выполняющих каких-либо функций. В целом вироиды представляют собой уникальную модель для исследования основ организации живых систем, многие из которых возникли на ранних этапах эволюции и остаются до сих пор не выявленными.

## Introduction

Viroids are highly structured circular single-stranded RNAs, which are able to replicate in infected organism and cause diseases from symptomless to lethal. Viroid genomes vary in size from 250 to 400 nucleotides: for example, it is 246 nt for Avocado sunblotch viroid (ASBVd) and Coconut cadang-cadang viroid, and 401 nt for Chrysanthemum chlorotic mottle viroid (CchMVd) (Srivastava, Prasad, 2020). Viroid genomes encode no proteins and the mechanisms of their replication and interactions with host cells are of great interest for specialists in phytopathology, molecular biology and molecular evolution.

Potato spindle tuber viroid (PSTVd) has been intensively investigated because of the adverse effects it has on potato yield. Solanum tuberosum is a vegetatively propagated crop, and thus it is especially vulnerable to viruses and viroids. PSTVd alone or in combination with viruses can decrease the yield of susceptible potato varieties from 40 to 70 % (Annenkov, 2000). The list of symptoms commonly includes leaf deformation, irregular tuber shape and development, slower sprouting, stunted phenotype, etc. Here we review recently published data on the PSTVd molecular genetics and the mechanisms mediating its specific pathological phenotypes.

## PSTVd genome structure, RNA population, and quasispecies

PSTVd strains can cause different symptoms. For instance, inoculation of tomato with PSTVd-Dahlia strain results in mild symptoms, while PSTVd-Intermediate strain causes a severe phenotype. Their genomes differ in nine positions, six of which are located in structured RNA parts (the left terminal domain and the pathogenicity domain). It was reported that mutation at pos. 42 decreases severity and viroid synthesis and mutation at pos. 64 affects stunting. In general, enrichment of mutations in genomic RNAs revealed positions with importance to symptom severity and viroid replication intensity (Kitabayashi et al., 2020). The viroid genome encodes no proteins and its replication depends on the host cellular machinery. The 3D-structure of the viroid RNA genome mediates its interaction with cellular proteins, for example, loop 27 (pos. 177–182) similar to a structural element in the 3′-UTR of animal histone mRNAs was found to be important for PSTVd replication and transport through host tissues.

Viroids are considered appropriate models for studying regulatory and catalytic RNAs as well as RNAmediated control of cellular processes. Interestingly, the PSTVd genome contains 17 G/U complementary interactions and some of them are conserved and functionally important for replication and systemic spreading throughout plant tissues (Wu J. et al., 2020). Artificial RNA constructs derived from viroid genomes can form circular molecules even in Saccharomyces cerevisiae, suggesting that their processing mechanisms are highly conservative (Friday et al., 2017).

Interestingly, viroid replication is error-prone, which results in a population of diverse genomic RNA molecules (“quasispecies”). The most frequent PSTVd genomic variants were analyzed at different time points after inoculation of tomato plants. It was found that at seven DAI only 25 % of the sequenced PSTVd genomes were identical to the master copy, but its frequency grew up to 70 % at 14 DAI and remained the same at 28 DAI (Adkar-Purushothama et al., 2020). It is likely that viroid replication produces a large variety of structural variants with different effects on host defense and the severity of disease symptoms. Viroids that replicated in plastids had higher mutation rates (1/800–1/1000 nucleotides) than those that replicated in nuclei (e.g., the mutation frequency for PSTVd varies between 1/3800 and 1/7000) (López-Carrasco et al., 2017).

Mutations in the viroid RNA genome can cause different effects on its replication and life cycle. Three engineered PSTVd genomes with small deletions or insertions were characterized (Więsyk et al., 2017). In two cases, the viroid lost the replication ability, while, in one case, it was still replicating, but its genome stability was decreased. Further analysis of inoculated tomato plants revealed frequent cases of reversion to the master copy and a variety of new stable genomic variants. It is likely that viroid genomes can evolve rapidly (Więsyk et al., 2017).

Viroids may be transmitted both vertically (through micro- or macrospores of infected plants) and horizontally (with pollen of infected plants). PSTVd is transmitted to future generations through macrospores, while some other viroids (e. g., tomato planta macho viroid, TPMVd) spread with pollen (Matsushita et al., 2018). The terminal left (TL) and pathogenicity (P) domains of the TPMVd genome were found to be responsible for pollen-mediated transmission (Yanagisawa et al., 2019). Interestingly, pollen grains from infected Petunia plants could infect tomato, i. e. viroid transmission does not require fertilization (Yanаgisawa, Matsushita, 2018).

## Pathogenesis mechanisms, plant defense mechanisms, and RNA interference

Inoculation of potato plants with PSTVd resulted in the accumulation of jasmonic acid in leaves, castasterone in leaves and roots, indole-3-acetic acid in tubers and no increase in salicylic or abscisic acids. In addition, viroid infection induced accumulation of reactive oxygen species (ROS) and enhanced the activity of antioxidants (Milanović et al., 2019). A metabolomic analysis revealed considerable changes in the content of 79 substances associated with 23 metabolic chains (Bagherian et al., 2016).

The molecular mechanisms associated with the severity of symptoms caused by different viroid strains remain underinvestigated. It is considered that symptoms depend on the host genotype and plant growing conditions (temperature, humidity, etc.). A comparison of the transcriptomes of tomato leaves after inoculation with either severe or mild PSTVd strains revealed more than 3000 DEGs; however, most of them were specific for the severe strain. Symptom severity were likely correlated with the expression of the genes coding for the С2С2GATA transcription factor and the growth regulatory factor (GRF) (Więsyk et al., 2020). Similar results were obtained for tomato roots inoculated with the mild and severe PSTVd strains: in addition to differences in expression between the genes controlling the induction of defense response, significant differences in expression were found between the genes for lignin biosynthesis and cell wall formation, and the genes of the auxin and cytokinin transduction pathways (Góra-Sochacka et al., 2019). Viroids produce neither proteins nor typical pathogen-associated molecular patterns (PAMP), and so the mechanisms of the induction of defense response remain unclear (Zheng et al., 2017; Nath et al., 2020).

The replication of viroids, their accumulation and traffic between plant tissues do not necessarily result in the development of disease symptoms. The severity of symptoms strongly depends on the viroid strain and host genotype. It is quite likely that some viroids infect plants without producing visible symptoms (it is possible that there are many undiscovered symptomless replicons persisting in the populations of host organisms). Interestingly, the disease symptoms may result from the RNA-interference induced by highly structured viroid genomic RNAs or replication intermediates. The viroid-derived siRNAs may target some host mRNAs and change the expression levels of the corresponding genes. If these genes participate in the control of plant development, physiological or biochemical processes, their suppression may result in the disease phenotype. 

PSTVd infection stunts potato growth, results in aberrations in leave and tuber morphology and decreased yield. It was found that PSTVd induces siRNAs related to the StTCP23 transcription factor (the teosinte branched1/Cycloidea/Proliferating cell factor). The StTCP23 mRNA 3′-UTR contains a 21-nucleotidelong segment complementary to the VMR (virulencemodulating region) of PSTVd strain RG1. Experimental suppression of StTCP23 with artificial microRNAs resulted in a potato phenotype similar to PSTVd disease symptoms. The functions of this gene are related to the gibberellic acid signal transduction pathway controlling plant growth and tuber development (Bao et al., 2019).

Flores et al. (2020) demonstrated that infected plants contain vd-sRNAs (viroid-derived small RNAs) able to interact with Argonaute proteins. PSTVd replication takes place in the nucleus and in this case vd-sRNAs appear at later stages of infection when systemic defense response is already a factor. Peach latent mosaic viroid (PLMVd) replicates in plastids and in this case vd-sRNAs appear at an early infection stage locally (Flores et al., 2020).

In another study (Adkar-Purushothama et al., 2018), one of the PSTVd-induced vd-sRNAs targeted the mRNA of tomato FRIGIDA-like protein 3. Tomato plants infected with severe strains of PSTVd are characterized by early flowering. Experimental suppression of FRIGIDA-like protein 3 results in a similar phenotype (Adkar-Purushothama, Perreault, 2018; Adkar-Purushothama et al., 2018).

RNA-interference is the mechanism controlling host defense against viruses and viroids. Experimental suppression of the Dicer 2 and Dicer 4 genes in tomato resulted in viroid accumulation and more severe symptoms. Interestingly, these proteins also take part in ROS generation and their absence interferes with the general defense response (Suzuki et al., 2019).

Eukaryotic mRNAs consist of the CDS and the 5′- and 3′-untranslated regions (UTRs). The 5′-UTR is responsible for translation initiation, while the 3′-UTR can influence mRNA cytoplasmic stability (Kochetov et al., 2002, 2004a, b; Kochetov, Sarai, 2004; Volkova, Kochetov, 2010; Ventoso et al., 2012). Self-complementary double-stranded RNAs (dsRNAs) are commonly used for engineered RNA-interference. Unlike CDS, most 3′-UTRs are not evolutionarily conserved and can be used as regions of choice for dsRNA-mediated selective gene suppression. Indeed, gene editing may be applied to remove the segment homologous to the viroid genome from the 3′-UTR regions of the host genes related to disease symptoms. Making viroid infection symptomless may strongly decrease yield loss and provides a new way for the molecular breeding of resistant cultivars (Kochetov et al., 2004b).

## Bioinformatics methods for viroid detection and analysis

NGS techniques have provided a way for a systemic large-scale analysis of transcriptomes and revealed a large variety of new viruses and viroids. Computational methods for viroid detection are commonly similar to those developed for viruses (Burger, Maree, 2015; Pecman et al., 2017)

It should be noted that the population of virus or viroid genomic molecules in the tissues of the infected organism may be heterogeneous because of frequent replication errors. These genomic variants are considered quasispecies (Brass et al., 2017). Thus, computational identification of viroids is frequently based on a combination of mapping reads to the known viroid sequences from databases and de novo sequence assembly. Viral RNAs may consist of a small part of the cellular transcriptome, and for that reason high coverage sequencing data are needed for a reliable detection of viroids – or special methods for viral RNA enrichment should be applied (Roossinck, 2012). In other words, the search for viruses and viroids in metatranscriptomes is relatively expensive. 

Computational analysis for viroid detection may include (Wu Q. et al., 2015):

- Analysis of transcriptomes to reveal and describe viral/viroid RNAs.
- Characterization of the population of viroid RNA molecules in tissues of an infected organism.
- Characterization of viroid RNA genomes in a natural or a model population of host plants.

To increase sensitivity, it seems reasonable to deplete mRNA libraries of fractions of small or ribosomal RNAs. A comparative analysis of these approaches on nine transcriptomes of infected plants demonstrated that the depletion of small RNAs in the libraries increased the sensitivity of detection of viroids and viruses with single-stranded DNA genomes, while the depletion of ribosomal RNAs was efficient for viruses with RNA genomes (Pecman et al., 2017). 

The Viral Surveillance and Diagnosis (VSD) pipeline was developed for viroid identification (Barrero et al., 2017). Short (21–24 nt) sequencing reads are taken as input. The pipeline consists of several modules (see Figure). The first module (a) assembles the genomes from short reads and performs adapter trimming, a quality check and removal of poorly assembled RNAs. SPAdes (Bankevich et al., 2012) and CAP3 (Huang, Madan, 1999), genome assembly tools, are used to select contigs longer than 40 nucleotides. The second module (b) contains programs for identification of transcripts corresponding to the host genome, viruses and viroids through a comparison with nucleotide and protein sequence databases with the aid of BLASTN and BLASTХ (Altschul et al., 1997). The third module (c) provides tools for prediction of new viroids. For this purpose, RNA contigs between 200 and 460 nucleotides are checked for the presence of 5′- and 3′-end overlaps characteristic of circular molecules. 

**Fig. 1.**
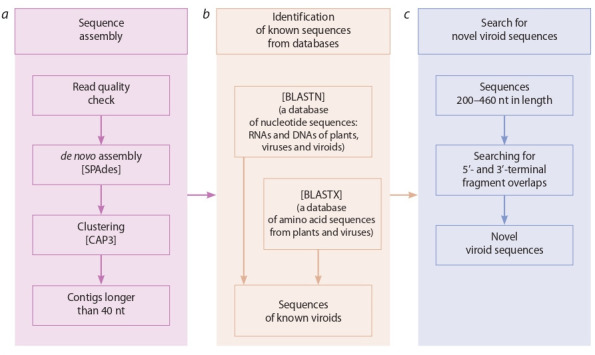
The VSD pipeline for identification of viral and viroid sequences in plant transcriptomic data. The pipeline consists of three modules: а, de novo sequence assembly; b, identification of the known sequences of plant viruses and viroids; c, the search for novel viroid sequences. Adapted from Barrero et al. (2017) Fig. 1.

The method of viroid identification on the basis of comparison with reference sequences was developed by Brass et al. (2017). It consists of modules for executing a standard protocol: removal of adapters and poly(A)-tails with PrinSeq (Schmieder, Edwards, 2011) and TRIMMOMATIC (Bolger et al., 2014) followed by quality checking and filtering with SEGEMEHL, and alignment onto the reference database (Otto et al., 2014). This tool supports identification of viroid quasispecies in RNA libraries. It was used for analysis of tomato cultivar ‘Heinz 1706’ transcriptomes bearing PSTVd strains QFA, C3 and AS1, ‘Rutgers’ transcriptomes with PSTVd strains M and I, and transcriptomes of four tomato cultivars infected with PSTVd strain RG. The results obtained were useful for evaluation of viroid evolutionary dynamics (Brass et al., 2017).

## Conflict of interest

The authors declare no conflict of interest.
